# Similarity-Based Virtual Screen Using Enhanced Siamese Multi-Layer Perceptron

**DOI:** 10.3390/molecules26216669

**Published:** 2021-11-03

**Authors:** Mohammed Khaldoon Altalib, Naomie Salim

**Affiliations:** 1 School of Computing, Universiti Teknologi Malaysia, Johor Bahru 81310, Malaysia; 2 Computer Science Department, Education for Pure Science College, University of Mosul, Mosul 41002, Iraq

**Keywords:** drug discovery, ligand-based virtual screen, similarity model, Siamese architecture, multi-layer perceptron (MLP)

## Abstract

Traditional drug development is a slow and costly process that leads to the production of new drugs. Virtual screening (VS) is a computational procedure that measures the similarity of molecules as one of its primary tasks. Many techniques for capturing the biological similarity between a test compound and a known target ligand have been established in ligand-based virtual screens (LBVSs). However, despite the good performances of the above methods compared to their predecessors, especially when dealing with molecules that have structurally homogenous active elements, they are not satisfied when dealing with molecules that are structurally heterogeneous. The main aim of this study is to improve the performance of similarity searching, especially with molecules that are structurally heterogeneous. The Siamese network will be used due to its capability to deal with complicated data samples in many fields. The Siamese multi-layer perceptron architecture will be enhanced by using two similarity distance layers with one fused layer, then multiple layers will be added after the fusion layer, and then the nodes of the model that contribute less or nothing during inference according to their signal-to-noise ratio values will be pruned. Several benchmark datasets will be used, which are: the MDL Drug Data Report (MDDR-DS1, MDDR-DS2, and MDDR-DS3), the Maximum Unbiased Validation (MUV), and the Directory of Useful Decoys (DUD). The results show the outperformance of the proposed method on standard Tanimoto coefficient (TAN) and other methods. Additionally, it is possible to reduce the number of nodes in the Siamese multilayer perceptron model while still keeping the effectiveness of recall on the same level.

## 1. Introduction

Drug discovery is a prolonged and complex process that culminates in the manufacture of new drugs. The biomolecular target is selected, and high-performance screening procedures are executed to identify bioactive chemicals for defined aims in traditional drug research and development. It is costly and time-consuming to produce high-performing research testing [1]. In truth, the chances of success are slim; approximately 1 out of every 5000 drug candidates is expected to be accepted and widely used at some point [2]. Increased computer capabilities, on the other hand, have enabled the screening of millions of chemical compounds at a reasonable speed and cost. The virtual screening methodology is a computerized method for scanning large libraries of small compounds for the most likely structures with the goal of developing medication [3,4,5]. Virtual screening (VS) is used in the early stages of drug development to identify the most promising lead compounds from large chemical libraries. The development of medications has been sped up in recent years thanks to virtual screening (VS). Virtual screening is divided into two types: structure-based virtual screening (SBVS) and ligand-based virtual screening (LBVS) [6]. The SBVS approaches strategies to look for indirect chemicals that are suitable for the binding site of the biological target. The molecular docking technique lies at the heart of SBVS approaches [7]. On the other hand, the LBVS approach is used constantly for the prediction of molecular properties and for measuring molecular similarity because the method to represent the molecules is easy and accurate. The necessity of applications of similarity searching comes from the importance of lead optimization in drug discovery programs, in which close neighbors are looking into an initial lead compound to find decent compounds [8,9,10].

Modern deep learning (DL) approaches have recently been presented in a variety of fields, and they have progressed in recent years, creating a new door for researchers. The hit of DL techniques benefits from the speedy growth of the DL algorithms and the progression of high-performance computing techniques. Moreover, DL techniques have slight generalization errors, which allow them to achieve credible results on certain benchmarks or competitive tests [11,12]. In addition, the Siamese network is frequently employed to solve image and text similarity problems. It has been utilized for more complex data samples, particularly heterogeneous data samples with a variety of dimensionality and type properties [13,14]. However, some studies reported that pruning the parameters makes the model of deep learning smaller in size, more memory-efficient, more power-efficient, and faster at inference. The whole idea of model pruning is to reduce the number of parameters without much loss in the accuracy of the model, which means cutting down parts of the network that contribute less or nothing to the network during inference [15,16,17].

Various techniques have been utilized in order to augment the retrieval effectiveness of similarity search algorithms. The use of 2D similarity algorithms has gained popularity. Estimating molecular similarity is based on the assumption that structurally similar molecules seem to be more likely to have similar characteristics than structurally different ones. Therefore, the objective of similarity searching is to identify molecules that are similar in structure to the consumer’s reference structures [18,19]. A number of coefficient techniques can be used to quantify the similarity/difference between molecule pairs. Many other studies tested the outcomes of various similarity coefficients, showing that the Tanimoto coefficient performed better than the others. As a result, in cheminformatics, the Tanimoto coefficient has become the most often used measure of chemical compound similarity [20,21,22]. Some experiments attempted to combine techniques from other fields. Such that they adapted the techniques from text information retrieval to employ in the cheminformatic domain to improve the similitude of molecular searching [23]. For example, the Bayesian inference network was based on text retrieval domains, it has been adapted and used in the similarity of molecular searches in virtual screening, and outperforms the Tanimoto technique [24,25]. Furthermore, reweighting approaches were employed to model document retrieval in the text field and modified in the cheminformatic area in the retrieval model [25,26,27]. Mohammed Al Dabagh (2017) improved the molecular similarity searching and molecular ranking of chemical compounds in LBVS using quantum mechanics physics theory principles [28]. Mubarak Hussien (2017) constructed a new similarity measure using current similarity measures by reweighting several bit-strings. Furthermore, the author offered ranking strategies for the development of a replacement ranking approach [29]. Deep belief networks (DBN) were used by Nasser, Majed, and colleagues (2021) to reweight molecular data wherein many descriptors were used, each reflecting separate relevant aspects, and combining all new features from all descriptors to create a new descriptor for similarity searches [30,31].

On the other hand, many studies have used deep learning methods as prediction or classification models. Some of them have used the DNN model to predict the activities of the selected compounds. Furthermore, other studies have reported that deep learning methods in Siamese architecture as a similarity model produce the best performance in many fields. For example, Jonas et al. (2016) used an LSTM Siamese neural network to calculate the similarity wherein the exponential Manhattan distance was used to measure the similarity between two sentences [32]. Jun Yu and Mengyan Li et al. (2020) used CNN Siamese architecture to determine whether two people are related, allowing missing persons to be reunited with their kin [33]. In the drug discovery domain, Devendra Singh Dhami et al. (2020) used images as an input in a Siamese convolutional network architecture to predict drug interactions in the drug discovery area [34]. Minji Jeon1 et al. (2019) proposed a method for calculating distance, utilizing MLP Siamese neural networks (ReSimNet) in structure-based virtual screen (SBVS) using cosine similarity [35].

Moreover, some early work in the pruning parameters domain used a gradual pruning scheme based on pruning all the weights in a layer less than some threshold (manually chosen) [12]. Blundell et al. (2015) introduced Bayes backpropagation for feedforward neural networks. This method gives the uncertainty in their predictions and reduces the model’s parameter count by ordering the weights according to their signal-to-noise-ratio and setting a certain percentage of the weights with the lowest ratios to 0 to prune these weights [15]. Louizos and Christos et al. (2017) used hierarchical priors to prune nodes instead of individual weights and also used the posterior uncertainties to determine the optimal fixed-point precision to encode the weights [36]. Chenglong Zhao et al. (2019) proposed a variational Bayesian scheme for pruning convolutional neural networks at the channel level. The variational technique is introduced to estimate the distribution of a newly proposed parameter; based on this, redundant channels can be removed from the model [37].

Despite the good performances of the above methods compared to their prior, especially when dealing with molecules that have homogenous active elements structural such as classes of molecules in the MDL Drug Data Report dataset (MDDR_DR2), the performances are not satisfied when dealing with molecules with structurally heterogeneous nature such as classes of molecules in the MDL Drug Data Report dataset (MDDR_DR3, MDDR_DR1) and maximum unbiased validation (MUV) dataset. In this paper, the Siamese multi-layer perceptron model will be used and enhanced in order to achieve the main purpose of this study for improving the performance of similarity searching, especially with molecules that are structurally heterogeneous. The following are the paper’s main contributions:

(1) The Siamese multi-layer perceptron will be enhanced by (a) using two distance layers and then a fuse layer that combines the results from two distance layers, with multiple layers added after the fusion layer to improve the similarity recall; (b) pruning nodes in the Siamese similarity model to reduce the number of parameters that contribute less or nothing to the network during inference.
(2) In comparison to the benchmark approach and previous studies, the suggested method outperformance in terms of results, especially when dealing with heterogeneous classes of molecules.

## 2. Materials and Methods

**Figure 1:**
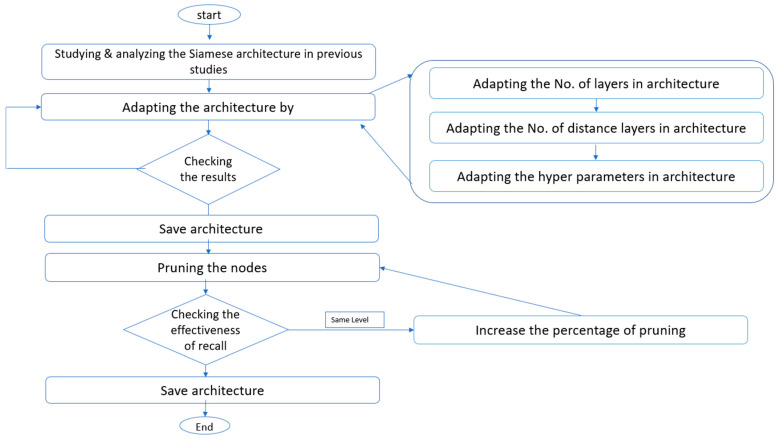
The flowchart of enhancing the Siamese multi-layer perceptron architecture.

A Siamese neural network includes two artificial neural networks that are the same, each able to handle the hidden input data representation, which have to be linked to a final layer using a distance layer to predict whether or not two vectors fall under the same group. The networks that make up the Siamese architecture are called twins since all the weights and biases are connected. This means that both networks are symmetric. During training, the two neural networks use both feedforward perceptron and error back-propagation. As a result, it has been applied to more complex data samples, particularly heterogeneous data samples with varying dimensionality and type properties [13]. In this paper, the Siamese multilayer perceptron (MLP) model will be enhanced; the flowchart of steps for enhancing the Siamese architecture is presented in Figure 1:

The steps to enhance the Siamese architecture of the multilayer perceptron include:

(1) Many models of Siamese architecture have been studied and analyzed in various domains, such as Minji Jeon1 et al. (2019) [35], Devendra Singh Dhami et al. (2020) [34] in the field of structure-based virtual screens, and Jonas et al. (2016) in the text field [32].
(2) All prior studies used one distance layer. In this paper, two distance layers are used, and then one fusion layer combines the results from distance layers. The reason for using more than one distance layer is to further improve the similarity measurements between molecules.
(3) Many layers have been added after the fusion layer to improve the retrieval recall.
(4) To acquire a good retrieval recall outcome, the model hyperparameters, such as the number of epochs and batch size, optimization, and the activation function, have been tuned.
(5) Finally, the nodes of the model that contribute less or nothing to the network during inference are pruned without having an effect on the effectiveness of the retrieval recall.

The architecture of the Siamese MLP similarity model and the mechanism of pruning the nodes will be explained in the following subsections.

### 2.1. Enhanced Siamese Multi-Layer Perceptron Similarity Model

The architecture of the Siamese MLP similarity model consists of two inputs, representing molecular descriptors (fingerprints), and has one output that represents the degree of similarity, meaning that the output has two classes; a value of (1) means high similarity and a value of (0) means high dissimilarity. In this model, the input layer has 1024 cells, each one connected to one feature of the molecular fingerprint, with each input layer connected to distance layers. Two distances were used; the first one was the Manhattan distance, which can be represented as [38]:

(1) $$d_{AB}=\left|f_{A}-f_{B}\right|\;$$

*d*_AB_: Manhatten distance

*f*_A_: feature of molecule’s query

*f*_B_: feature of molecule’s dataset

And the second distance was exponential Manhattan distance and can be given as [32]:

(2) $$E_{AB}=exp\left(-\left|f_{A}-f_{B}\right|\right)$$

*E*_AB_: exponential Manhatten distance

*f*_A_: feature of molecule’s query

*f*_B_: feature of molecule’s dataset

**Figure 2:**
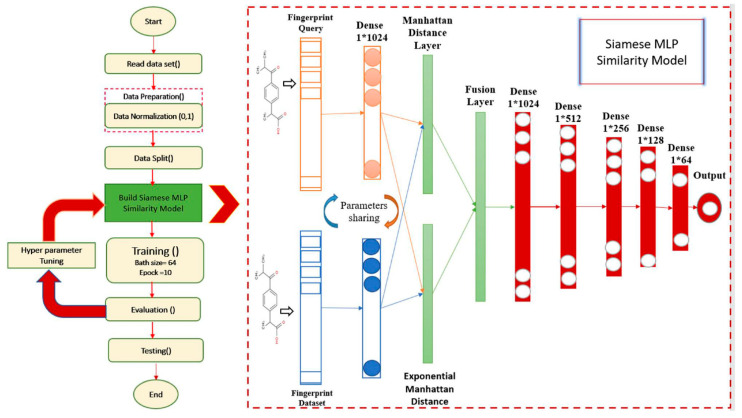
Enhanced Siamese MLP similarity model.

A fusion layer was then added between two distance layers—Manhattan and Exponential Manhattan—and was the reason for using more than one similarity distance to enhance the measures of similarity between molecules. The ReLU activation function has been used for all layers except the last one, in which the sigmoid activation function has been used. Moreover, the RMSprop optimizer has been used and the loss function was (binary_crossentropy); and the batch size was 256. Figure 2 demonstrates the architecture of the enhanced Siamese MLP similarity model.

### 2.2. Nodes or Neurons Pruning

**Figure 3:**
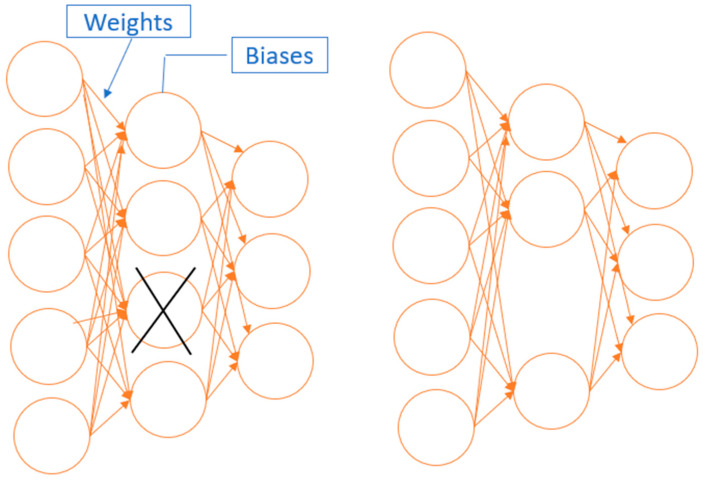
The idea of node pruning.

As deep neural networks contain more layers, we must multiply multiple floating-point integers together, which takes a long time to train and infer and consumes a lot of computing resources. The problem mentioned above can be solved in a number of ways, including weight sharing, pruning, quantization, and so on. The goal of model pruning is to reduce the number of parameters while maintaining model correctness, which entails pruning parts of the network that give little or no information to the network during inference. As a result, models are smaller in size, and more memory-efficient, power-efficient, and faster at inference with low accuracy loss [16,39]. Weight pruning and node pruning are the two most common methods of pruning. In weight pruning, individual weights are ranked in a weight matrix W based on their magnitude (or any other criterion), and the smallest k percent of the weights are set to zero in weight trimming. This corresponds to deleting connections between nodes in different layers. However, in node pruning, the columns that represented nodes in weight matrix are set to zero, in effect deleting the corresponding output neuron. Here, nodes are ranked according to their magnitude (or any other criterion), and the smallest k percent of each node is set to zero. Pruning nodes will be employed in this research. Figure 3 demonstrates the idea of node pruning.

Each node is represented by a column of values in the weights matrix; the mean and variance of the column (node) are evaluated, and then the signal-to-noise ratio is calculated [15], the formula for which is:

(3) $$signal\_to\_noise\;ratio=\frac{\left|\mu_{i}\right|}{\sigma_{i}}$$

*μ*: mean of column

*σ*: variance of column

*i*: the sequence of a column in the weight matrix

## 3. Experimental Design

### 3.1. Datasets

The MDL drug data report (MDDR) [40], maximum unbiased validation (MUV) [41], and directory of useful decoys (DUD) [42] were used in the experiments. These datasets are the most common cheminformatics datasets, and these datasets have recently been used by our study community. All molecules in the MDDR dataset were converted to fingerprints using the ECFC 4 descriptor. The screening studies were carried out with ten reference structures randomly selected from each activity class. Three 102,516-molecule datasets have been chosen (MDDR-DS1, MDDR-DS2, and MDDR-DS3). The MDDR-DS1 is divided into 11 activity groups, some of which have structurally homogeneous active elements and others which have structurally heterogeneous (i.e., structurally different) active elements. MDDR-DS2 contains ten homogeneous activity classes, whereas MDDR-DS3 contains ten heterogeneous activity classes. All of the datasets are described in Table 1, Table 2 and Table 3. Each row of a table includes the activity class, the number of molecules belonging to the class, as well as a diversity of groups, which were measured as the average similarity of Tanimoto, computed by ECFC 4 for all pairs of molecules. As shown in Table 4, Rohrer and Baumann recorded the second data collection (MUV) in this study. This data collection contains 17 interaction groups, each of which has up to 30 active and 15,000 inactive molecules. The class composition of this dataset shows that it contains classes with a lot of variety or processes that are more heterogeneous. The last group of data used in this study is the Useful Decoys Directory (DUD), which was recently compiled for docking methods as a benchmark dataset. It was introduced by Huang et al. (2006) and was recently used in both molecular and molecular virtual screening [43]. Twelve DUD subsets with 704 active compounds and 25,828 decoys were used in this study, as shown in Table 5.

### 3.2. Evaluation Measures of the Performance

The following criteria are used to assess the efficacy of the suggested method:

1. The first method is to look for active chemical compounds in the top 1% and 5% of the scored test set and calculate the recall value. This metric has been employed in a number of previous approaches [27,28,30,31,44,45,46,47,48].
2. Comparison method: the second method is to compare current techniques that may be utilized to evaluate the proposed model’s findings. These techniques include the following:
  (a) TAN: the Tanimoto similarity coefficient has been the search benchmark method in LBVS for many years. The Tanimoto coefficient is used in its continuous form for similarities. It has been utilized in the datasets DS1, DS2, DS3, MUV, and DUD.
  (b) BIN: the second technique is the Bayesian inference network, which used the ECFC4 descriptors in datasets DS1, DS2, DS3, and MUV. This is another way of comparing the results in the similarity model of molecular fingerprints in LBVS [24].
  (c) SQB: the third method is quantum similarity search SQB in the MDDR dataset (DS1, DS2, DS3, and MUV) for the ECFC4 descriptor. This method utilizes a quantum mechanics approach as the model of similarity searching in LBVS [28].
  (d) SDBN: the last technique is the deep belief networks, used to reweight the chemical characteristics, where ECFC-4, EPFP-4, and ECFP-4 descriptors were analyzed using the stack of deep belief networks technique on the MDDR dataset (DS1, DS2, DS3) [31].
3. The Kendall W concordance test is another important metric that could be used to measure the performance of the suggested techniques and rank the similarity methods. The concordance coefficient is a measure of agreement among raters. In the Kendall W test, each case represents a judge or rater, while each variable represents the thing or person being assessed. The domain of a Kendall W test score is between 0 and 1. If the test score is 0, this means no agreement; if the test score 1, this means complete agreement. Assume the object (i) is considered as the similarity method, (ranked objects) is given the rank $r_{ij}$ by the raters j (activity class), where there are in total (*n*) objects and (*m*) raters. Then, the total rank (*R*) given to object (i) is [49]:
  (4) $${ℜ}_{i}=\sum_{j=i}^{m}r_{ij}$$
  Then, the mean value (Ŕ) is calculated by these total rankings as:
  (5) $$\bar{ℜ}=\frac{1}{2}m\left(n+1\right)$$
  Then, the sum of squared deviations (δ) is calculated as:
  (6) $$\delta =\sum_{i=i}^{n}{\left({ℜ}_{i}-\bar{ℜ}\right)}^{2}$$
  Then, the Kendall W test is calculated as:
  (7) $$W=\frac{12\delta }{m^{2}\left(n^{3}-n\right)}$$

The results of this test are the Kendall coefficient (between 0 and 1) and significance level (*p*-value); if the *p*-value is less than 0.05, the result is considered significant, and the similarity methods can be ranked.

## 4. Results and Discussion

The experimental results from the MDDR-DS1, MDDR-DS2, MDDR-DS3, MUV, and DUD datasets, for the ECFC-4 descriptor, are provided in Table 6, Table 7, Table 8, Table 9, Table 10, Table 11, Table 12, Table 13, Table 14 and Table 15, respectively, using 1% and 5% cut-offs. These tables show the results of the enhanced Siamese MLP similarity model compared to the benchmark TAN, as well as earlier studies BIN, SQB, and SDBN for MDDR datasets, BIN and SQB for MUV datasets, and SQB for DUD datasets. Every row in the tables displays the recall for the top 1% and 5% of the activity class, with the best recall rate shaded in each row. The mean rows in the tables represent the average for all activity classes, whereas the rows with shaded cells represent the total number of shaded cells for each technique throughout the whole range of activity classes.

When comparing the MDDR-DS1 recall results for the top 1% and 5% in Table 6 and Table 7, the suggested enhanced Siamese MLP technique was clearly superior to the benchmark TAN method and prior studies BIN, SQB, and SDBN in terms of the mean and number of shaded cells. The suggested technique has the highest mean value (30.69) in Table 6, followed by SDBN, BIN, SQB, and lastly, TAN methods. In the suggested approach, the shaded cells have a value of (7). The suggested approach has the highest mean value (50.463) in Table 7, followed by SDBN, BIN, SQB, and lastly TAN methods. In the suggested technique, shaded cells have a value of (9).

Furthermore, the MDDR-DS2 recall values obtained at the top 1%, as shown in Table 8, demonstrate that the suggested Siamese MLP technique outperforms the benchmark TAN method. In view of the number of shaded cells, the MLP approach produced the best retrieval recall results, and the suggested method’s mean value is extremely close to that of prior research. However, by comparison, The MDDR-DS2 recall values obtained at the top 5% in Table 9 clearly shows that the suggested Siamese MLP approach outperforms the benchmark TAN method only. In terms of the mean and number of shaded cells, the BIN approach produced the best retrieval recall results. Next, the second is SQB, SDBN, and finally, TAN in view of the mean value.

In addition, the MDDR-DS3 recall values recorded at the top 1% and 5% in Table 10 and Table 11, respectively, show that the proposed enhanced Siamese MLP method is obviously superior to the benchmark TAN method and methods from other studies. Likewise, in Table 10, the proposed method gave the best retrieval recall results in view of the mean and number of shaded cells, compared to prior studies and benchmark TAN, followed by SDBN, BIN, SQB, and finally, TAN methods. By comparison, in Table 11, the suggested enhanced Siamese MLP method was obviously superior to the benchmark TAN method and other studies. The second one is SDBN, followed by TAN, BIN, and SQB.

Moreover, the MUV dataset recall values recorded at the top 1% and 5% in Table 12 and Table 13, respectively, show that the proposed enhanced Siamese MLP method is obviously superior to the benchmark TAN method and other studies. Likewise, in Table 12, the proposed method gave the best retrieval recall results in view of the mean and number of shaded cells, compared to the TAN method and methods from other studies, followed by BIN, SQB, and finally, TAN methods. However, by comparison, in Table 13, the proposed enhanced Siamese MLP method was obviously superior to the benchmark TAN method and methods from other studies. Next, the second one is BIN, followed by SQB and TAN.

Moreover, the DUD dataset recall values recorded at the top 1% and 5% in Table 14 and Table 15, respectively, show that the proposed enhanced Siamese MLP method is obviously superior to the benchmark TAN method and methods from other studies. Likewise, in Table 14, the proposed method gave the best retrieval recall results in view of the mean and number of shaded cells, compared to the previous study and benchmark TAN. Furthermore, in Table 15, the proposed enhanced Siamese MLP method was obviously superior to the benchmark TAN method and the previous study SQB.

**Figure 4:**
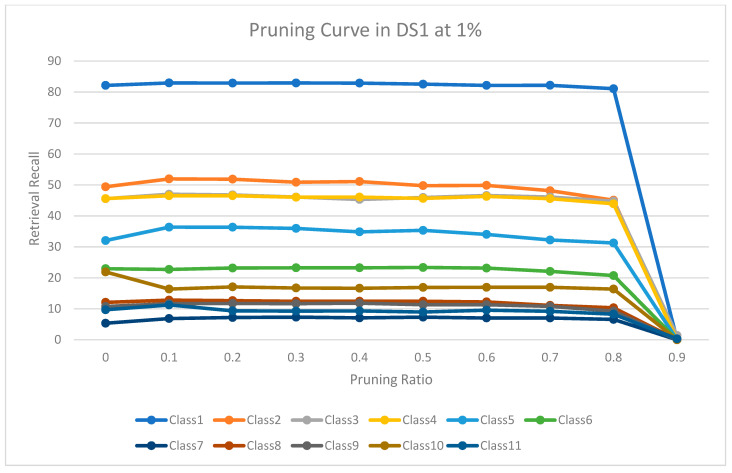
The level of retrieval recall values at different percentages of pruning at the top 1% in MDDR-DR1.

**Figure 5:**
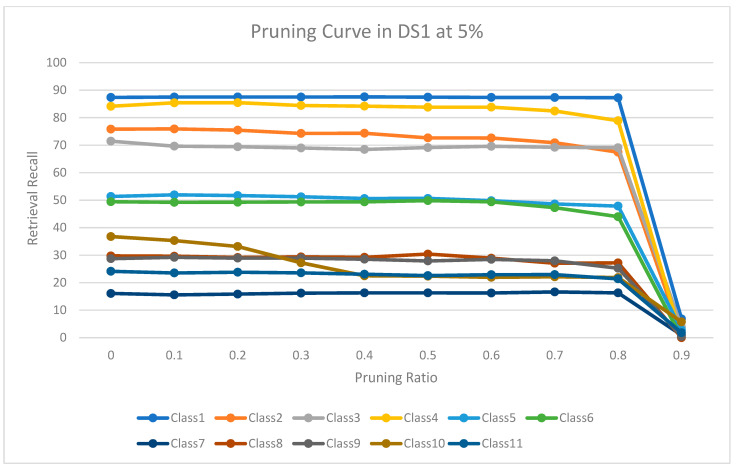
The level of retrieval recall values at different percentages of pruning at the top 5% in MDDR-DR1.

**Figure 6:**
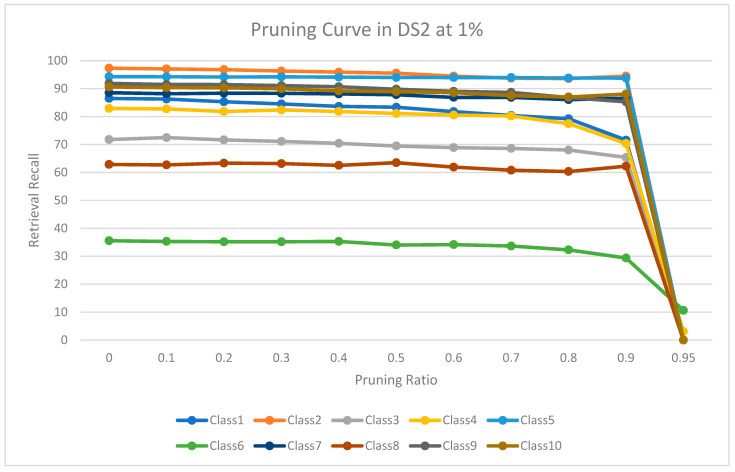
The level of retrieval recall values at different percentages of pruning at the top 1% in DDR-DR2.

**Figure 7:**
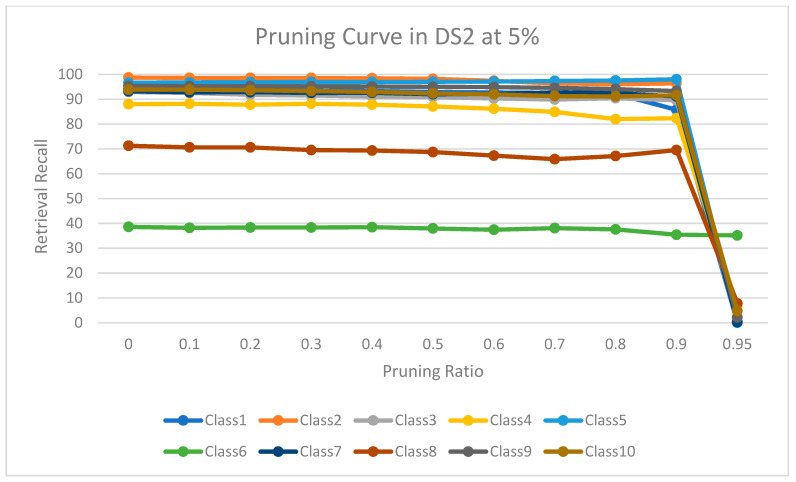
The level of retrieval recall values at different percentages of pruning at the top 5% in MDDR-DR2.

**Figure 8:**
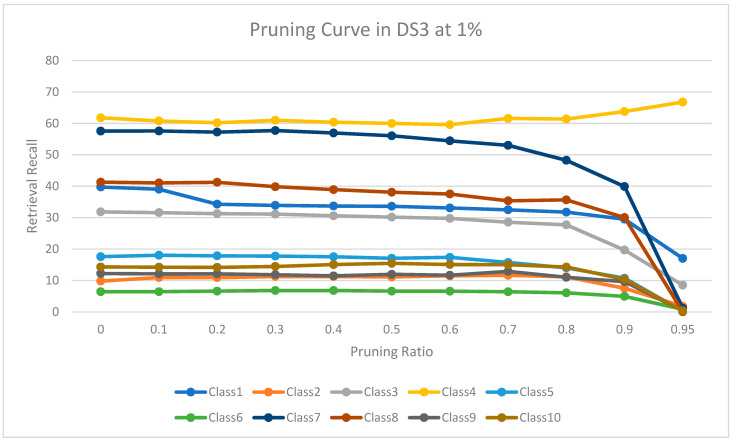
The level of retrieval recall values at different percentages of pruning at the top 1% in DR3-MDDR.

**Figure 9:**
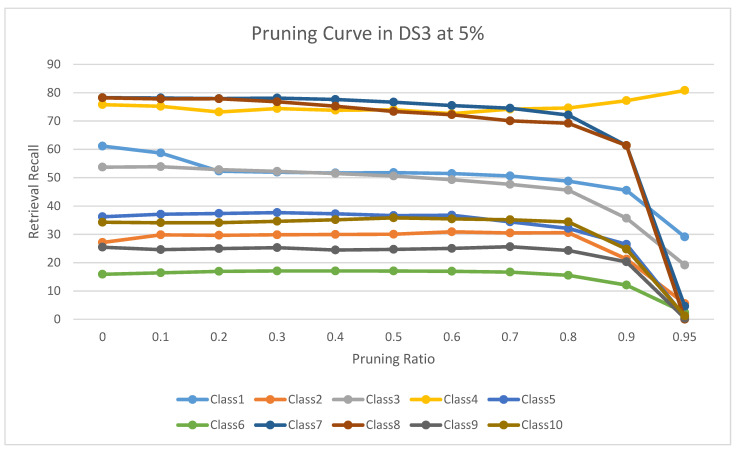
The level of retrieval recall values at different percentages of pruning at the top 5% in DR3-MDDR.

**Figure 10:**
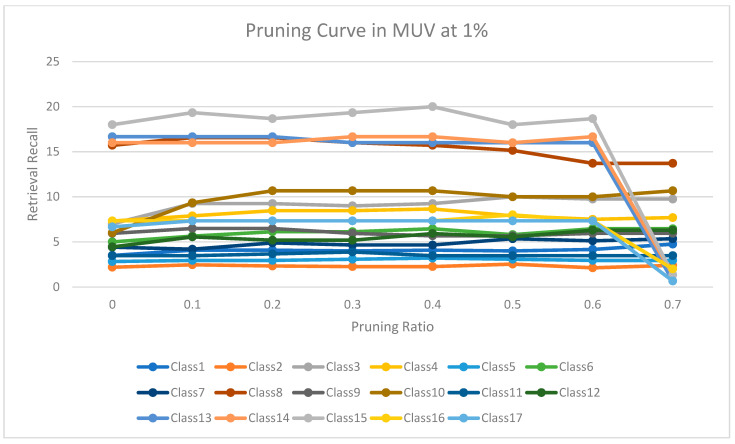
The level of retrieval recall values at different percentages of pruning at the top 1% in MUV dataset.

**Figure 11:**
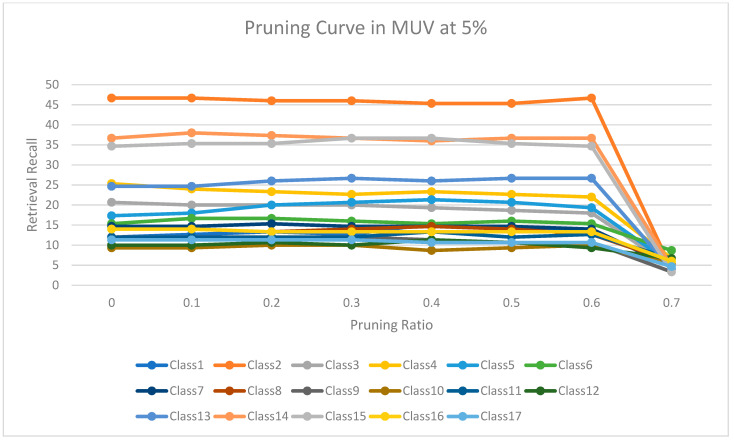
The level of retrieval recall values at different percentages of pruning at the top 5% in MUV dataset.

**Figure 12:**
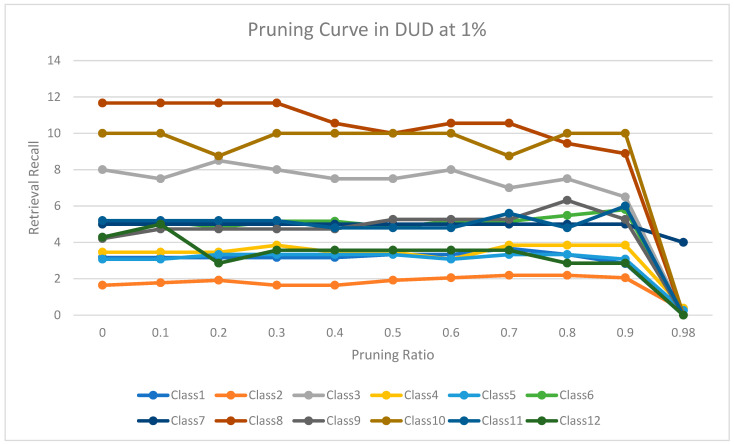
The level of retrieval recall values at different percentages of pruning at the top 1% in DUD dataset.

**Figure 13:**
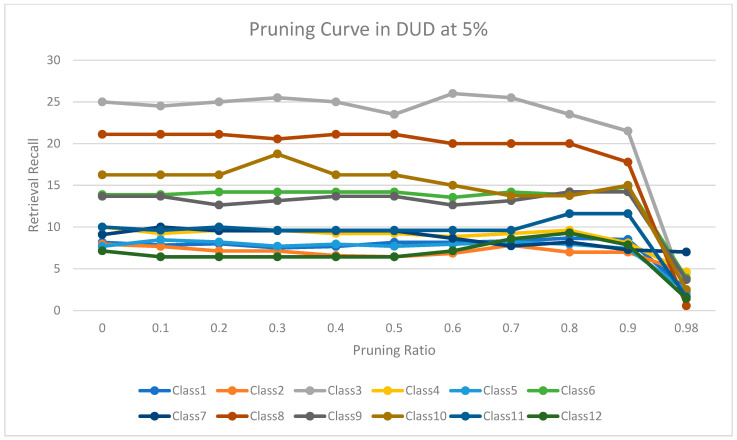
The level of retrieval recall values at different percentages of pruning at the top 5% in DUD dataset.

The experimental results for pruning nodes on MDDR-DS1, MDDR-DS2, MDDR-DS3, MUV, and DUD datasets are shown in Figure 4, Figure 5, Figure 6, Figure 7, Figure 8, Figure 9, Figure 10, Figure 11, Figure 12 and Figure 13, respectively. In these figures, the x axis represents the pruning ratio starting from 0% and ending with 90%, 95%, 95%, 70%, and 98% in DS1, DS2, DS3, MUV, and DUD datasets, respectively. They y axis represents the level of retrieval recall values for each class in the dataset. The classes of molecules are represented as color lines. The tables that contain on pruning ratio of recall values for each dataset are available as Supplementary Materials.

Figure 4 shows the level of the retrieval recall values at different pruning ratios for each class at the top 1% in DS1. We note that the recall values of most classes remain the same until they reach 80% of the pruning ratio, while some classes increased slightly, such as class 7, and decreased by a little in others, such as classes 2 and 9. Furthermore, Figure 5 shows the level of retrieval recall values at different pruning ratios for each class at the top 5% in DS1. The recall values of most classes remain the same values until they reach 80% of the pruning ratio, while some classes decreased, such as classes 2 and 10.

Figure 6 shows the level of retrieval recall values at different pruning ratios for each class at the top 1% in DS2. We note that the recall values of most classes remain the same until they reach 90% of the pruning ratio, while some classes increased slightly, such as classes 5 and 8, and decreased by a little in others, such as classes 1, 3, and 4. Furthermore, Figure 7 shows the level of retrieval recall values at different pruning ratios for each class at the top 5% in DS2. The recall values of most classes remain the same until they reach 90% of the pruning ratio, except for class 6, which remains until more than 95% of the pruning ratio, while some classes decreased by a little, such as class 4, or increased slightly, such as class 5.

Figure 8 shows the level of retrieval recall values at different pruning ratios for each class at the top 1% in DS3. We note that the recall values of most classes remain the same until they reach 80% of the pruning ratio, while some classes increased slightly, such as class 4, a more than 95% pruning ratio, and decreased by a little in others, such as classes 1, 7, and 8. Furthermore, Figure 9 shows the retrieval recall values at different pruning ratios for each class at the top 5% in DS3. The recall values of most classes remain the same until they reach 80% of the pruning ratio, except for class 4, which increased until more than 95%, while some classes decreased by a little, such as classes 1, 3, 7, and 8.

Figure 10 shows the level of retrieval recall values at different pruning ratios for each class at the top 1% in MUV. We note that the recall values of most classes remained the same until they reached 60% of the pruning ratio, while some classes increased slightly, such as classes 3 and 10, and decreased by a little in others, such as class 8. Moreover, Figure 11 shows the retrieval recall values at different pruning ratios for each class at the top 5% in MUV. The recall values of most classes remained the same until they reached 60% of the pruning ratio, while some classes increased slightly, such as classes 1, 3 and 5, and decreased by a little in others, such as classes 3 and 4.

Furthermore, Figure 12 shows the level of retrieval recall values at different pruning ratios for each class at the top 1% in DUD. We note that the recall values of most classes remained the same until they reached 80% of the pruning ratio, while some classes increased slightly, such as classes 4,6, and 9, and decreased by a little in others, such as classes 3, 8, and 12. Moreover, Figure 13 shows the retrieval recall values at different pruning ratios for each class at the top 5% in DUD. The recall values of most classes remained the same until they reached 80% of the pruning ratio, while some classes increased slightly, such as classes 11 and 12, and decreased by a little in others, such as classes 3 and 10.

**Figure 14:**
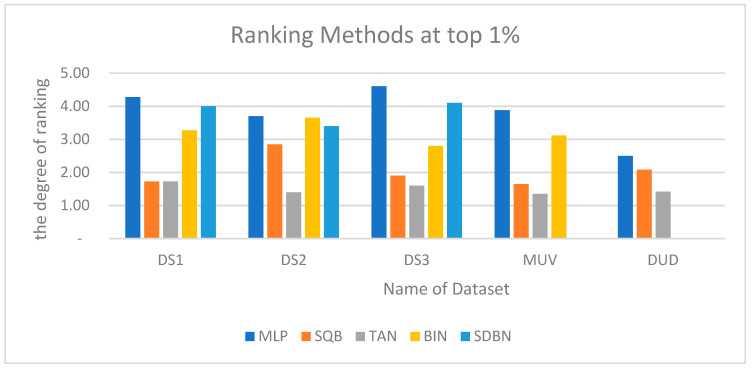
Ranking of enhanced Siamese multilayer perceptron method based on TAN, BIN, SQB, and SDBN using Kendall W test results for DS1, DS2, DS3, MUV, and DUD at top 1%.

**Figure 15:**
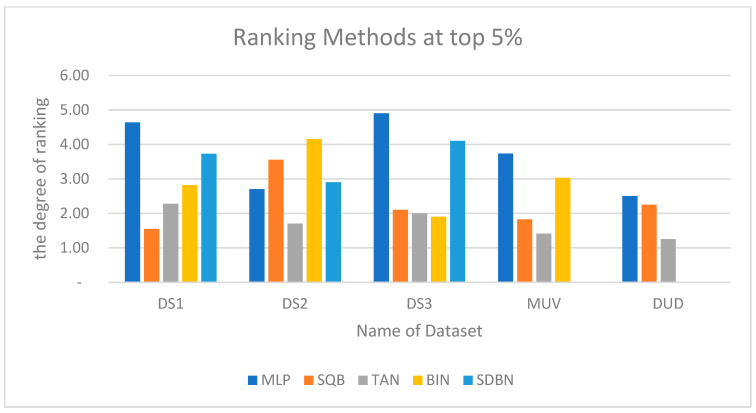
Ranking of enhanced Siamese multilayer perceptron method based on TAN, BIN, SQB, and SDBN using Kendall W test results for DS1, DS2, DS3, MUV, and DUD at top 5%.

Moreover, the Kendall W concordance test has been used; Table 16 shows the ranking of the enhanced Siamese multilayer perceptron method based on previous studies of TAN, BIN, SQB, and SDBN using Kendall W test results for MDDR-DS1, MDDR-DS2, MDDR-DS3, MUV, and DUD at the top 1% and 5%. The first method is the benchmark method, which is the Tanimoto coefficient TAN; the second method is Bayesian inference [24]; the third method is quantum similarity search SQB-Complex [28]; the last method is multi-descriptor-based on Stack of deep belief networks SDBN [31]. The results of the Kendall W test of the top 1% for all used datasets show that the values of associated probability (*p*) are less than 0.05. This indicates that the enhanced Siamese multilayer perceptron method is significant in the top 1% for all cases. As a result, the overall ranking of all methods indicates that the enhanced Siamese multilayer perceptron method is superior to previous studies and benchmark TAN. The overall ranking for methods showed that MLP has the top ranks among other methods. This is the same as with the results of the Kendall W test of the top 5%; the results show that the values of associated probability (p) are less than 0.05. This indicates that the enhanced Siamese multilayer perceptron method is significant in the top 5%. As a result, the overall ranking of all methods indicates that the enhanced Siamese multilayer perceptron method is superior to previous studies for all datasets and the overall ranking for the method showed that Siamese multilayer perceptron method has the top ranks among other methods, except in DR2, in which the BIN method was better than MLP. Figure 14 and Figure 15 show the ranking of the enhanced Siamese multilayer perceptron method based on TAN, BIN, SQB, and SDBN using the Kendall W test results for DS1, DS2,DS3, MUV, and DUD at the top 1% and 5%.

## 5. Conclusions

Many techniques for capturing the biological similarity between a test compound and a known target ligand in LBVS have been established. LBVS is based on the premise that the target-binding behavior of related property compounds will be related. In spite of the good performances of the above methods compared to their prior, especially when dealing with molecules that have structurally homogenous active elements, the performances are not satisfied when dealing with molecules that are structurally heterogeneous. The main goal of this research was to improve the retrieval effectiveness of the similarity model, especially with molecules that are structurally heterogeneous. In this study, the Siamese multilayer perceptron similarity model has been enhanced by using two distance layers with a fuse layer that combines the results from two distance layers, and then multiple layers were added after the fusion layer, followed by pruning of the nodes that contribute less or nothing to the network during inference according to their signal-to-noise ratio. The results showed that the significance of the proposed method obviously outperformed the standard Tanimoto coefficient (TAN) and previous studies (BIN, SQB, and SDBN) at the top 1% and 5% for MDDR-DS1, MDDR-DS3, DUD, and MUV, which include heterogeneous classes. Additionally, the proposed method has the top rank for the top 1% MDDR-DS2, which include homogeneous classes. Besides that, it is possible to reduce the number of nodes in the Siamese multilayer perceptron model while still keeping the effectiveness of recall on the same level when pruning 60% of nodes in MUV, 90% in DS2, and 80% in DS1, DS3, and DUD. Multiple molecular descriptors will be tested in this proposed method as future work.

## Figures and Tables

**Table 1:** The MDDR-DS1 structure activity classes.

Activity Index	Active Molecules	Activity Class	Pairwise Similarity
31420	1130	Renin inhibitors	0.290
31432	943	Angiotensin II AT1 antagonists	0.229
37110	803	Thrombin inhibitors	0.180
71523	750	HIV protease inhibitors	0.198
42731	1246	Substance P antagonists	0.149
07701	395	D2 antagonists	0.138
06245	359	5HT reuptake inhibitors	0.122
78374	453	Protein kinase C inhibitors	0.120
06235	827	5HT1A agonists	0.133
06233	752	5HT3 antagonist	0.140
78331	636	Cyclooxygenase inhibitors	0.108

**Table 2:** The MDDR-DS2 structure activity classes.

Activity Index	Active Molecules	Activity Class	Pairwise Similarity
07707	207	Adenosine (A1) agonists	0.229
42710	111	CCK agonists	0.361
31420	1130	Renin inhibitors	0.290
64200	113	Cephalosporins	0.322
64100	1346	Monocyclic lactams	0.336
64500	126	Carbapenems	0.260
64220	1051	Carbacephems	0.269
75755	455	Vitamin D analogous	0.386
75755	455	Vitamin D analogous	0.386
07708	156	Adenosine (A2) agonists	0.305

**Table 3:** The MDDR-DS3 structure activity classes.

Activity Index	Active Molecules	Activity Class	Pairwise Similarity
09249	900	Muscarinic (M1) agonists	0.111
31281	106	Dopamine -hydroxylase inhibitors	0.125
12464	505	Nitric oxide synthase inhibitors	0.102
71522	700	Reverse transcriptase inhibitors	0.103
43210	957	Aldose reductase inhibitors	0.119
12455	1400	NMDA receptor antagonists	0.098
75721	636	Aromatase inhibitors	0.110
78351	2111	Lipoxygenase inhibitors	0.113
78348	617	Phospholipase A2 inhibitors	0.123
78331	636	Cyclooxygenase inhibitors	0.108

**Table 4:** MUV structure activity classes.

Activity Index	Active Molecules	Activity Class	Pairwise Similarity
66	30	S1P1 rec. (agonists)	0.117
644	30	Rho-Kinase2 (inhibitors)	0.122
600	30	SF1 (inhibitors)	0.123
689	30	Eph rec. A4 (inhibitors)	0.113
652	30	HIV RT-RNase (inhibitors)	0.099
712	30	HSP 90 (inhibitors) 30	0.106
692	30	SF1 (agonists)	0.114
733	30	ER-b-Coact. Bind. (inhibitors)	0.114
713	30	ER-a-Coact. Bind. (inhibitors)	0.113
810	30	FAK (inhibitors)	0.107
737	30	ER-a-Coact. Bind. (potentiators)	0.129
846	30	FXIa (inhibitors)	0.161
832	30	Cathepsin G (inhibitors)	0.151
858	30	D1 rec. (allosteric modulators)	0.111
852	30	FXIIa (inhibitors)	0.150
548	30	PKA (inhibitors)	0.128
859	30	M1 rec. (allosteric inhibitors)	0.126

**Table 5:** DUD structure activity classes, where $N_{a}$ denotes the number of active compounds, and N_dec_ denotes the number of decoys.

No.	Dataset	Active and Inactive	
		N_dec_	N_a_
1	FGFR1T	4550	120
2	FXA	5745	146
3	GART	879	40
4	GBP	2140	52
5	GR	2947	78
6	HIVPR	2038	62
7	HIVRT	1519	43
8	HMGA	1480	35
9	HSP90	979	37
10	MR	636	15
11	NA	1874	49
12	PR	1041	27
total	-	25,828	704

**Table 6:** Retrieval results of top 1% for MDDR-DS1 dataset for (ECFC_4) descriptor.

DS1	Previous Studies				Proposed Methods
Retrieval Result 1%					
Activity Index	TAN	BIN	SQB	SDBN	MLP
31420	69.69	74.08	73.73	74.21	82.1416
71523	25.94	28.26	26.84	27.97	49.4118
37110	9.63	26.05	24.73	26.03	45.5639
31432	35.82	39.23	36.66	39.79	45.5957
42731	17.77	21.68	21.17	23.06	32.0546
6233	13.87	14.06	12.49	19.29	22.9708
6245	6.51	6.31	6.03	6.27	5.36313
7701	8.63	11.45	11.35	14.05	12.0918
6235	9.71	10.84	10.15	12.87	10.7767
78374	13.69	14.25	13.08	17.47	21.9196
78331	7.17	6.03	5.92	9.93	9.70199
Mean	19.86	22.93	22.01	24.63091	30.69
Shaded cells	1	0	0	3	7

**Table 7:** Retrieval results of top 5% for MDDR-DS1 dataset for (ECFC_4) descriptor.

DS1	Previous Studies				Proposed Methods
Retrieval Result 5%					
Activity Index	TAN	BIN	SQB	SDBN	MLP
31420	83.49	87.61	87.22	89.03	87.3628
71523	48.92	52.72	48.70	65.17	75.8289
37110	21.01	48.20	45.62	41.25	71.4536
31432	74.29	77.57	70.44	79.87	84.1489
42731	29.68	26.63	24.35	31.92	51.3644
6233	27.68	23.49	20.04	29.31	49.443
6245	16.54	14.86	13.72	21.06	16.0894
7701	24.09	27.79	26.73	28.43	29.7449
6235	20.06	23.78	22.81	27.82	28.7379
78374	20.51	20.20	19.56	19.09	36.7857
78331	16.20	11.80	11.37	16.21	24.1391
Mean	34.77	37.70	35.51	40.83273	50.463
Shaded cells	0	0	0	2	9

**Table 8:** Top 1% retrieval results for MDDR-DS2 dataset for descriptor (ECFC 4).

DS2	Previous Studies				Proposed Methods
Retrieval Result 1%					
Activity Index	TAN	BIN	SQB	SDBN	MLP
7707	61.84	72.18	72.09	83.19	86.4706
7708	47.03	96.00	95.68	94.82	97.3077
31420	65.10	79.82	78.56	79.27	71.7699
42710	81.27	76.27	76.82	74.81	82.9091
64100	80.31	88.43	87.80	93.65	94.2769
64200	53.84	70.18	70.18	71.16	35.5696
64220	38.64	68.32	67.58	68.71	88.5333
64500	30.56	81.20	79.20	75.62	62.8571
64350	80.18	81.89	81.68	85.21	91.8557
75755	87.56	98.06	98.02	96.52	90.5727
Mean	62.63	81.24	80.76	82.296	80.21226
Shaded cells	0	3	0	1	6

**Table 9:** Top 5% retrieval results for MDDR-DS2 dataset for descriptor (ECFC 4).

DS2	Previous Studies				Proposed Methods
Retrieval Result 5%					
Activity Index	TAN	BIN	SQB	SDBN	MLP
7707	70.39	74.81	74.37	73.9	94.2157
7708	56.58	99.61	99.61	98.22	98.7179
31420	88.19	95.46	94.88	95.64	92.9381
42710	88.09	92.55	91.09	90.12	88
64100	93.75	99.22	99.03	99.05	96.6615
64200	77.68	99.2	99.38	93.76	38.6076
64220	52.19	91.32	90.62	96.01	93.2381
64500	44.8	94.96	92.48	91.51	71.2698
64350	91.71	91.47	90.78	86.94	95.3608
75755	94.82	98.35	98.37	91.6	93.8767
Mean	75.82	93.70	93.06	91.675	86.28862
Shaded cells	0	4	3	2	2

**Table 10:** Top 1% retrieval results for MDDR-DS3 dataset for descriptor (ECFC 4).

Ds3	Previous Studies				Proposed Methods
Retrieval Result 1%					
Activity Index	TAN	BIN	SQB	SDBN	MLP
9249	12.12	15.33	10.99	19.47	39.7556
12455	6.57	9.37	7.03	13.29	9.8
12464	8.17	8.45	6.92	12.91	31.84
31281	16.95	18.29	18.67	23.62	61.8
43210	6.27	7.34	6.83	14.23	17.5789
71522	3.75	4.08	6.57	11.92	6.42857
75721	17.32	20.41	20.38	29.08	57.5667
78331	6.31	7.51	6.16	11.93	41.3
78348	10.15	9.79	8.99	9.17	12.2
78351	9.84	13.68	12.5	18.13	14.3024
Mean	9.75	11.43	10.50	16.375	29.257217
Shaded cells	0	0	0	3	7

**Table 11:** Top 5% retrieval results for MDDR-DS3 dataset for descriptor (ECFC 4).

Ds3 Retrieval Result 5%	Previous Studies				Proposed Methods
Activity Index	TAN	BIN	SQB	SDBN	MLP
9249	24.17	25.72	17.8	31.61	61.1556
12455	10.29	14.65	11.42	16.29	27.1429
12464	15.22	16.55	16.79	20.9	53.72
31281	29.62	28.29	29.05	36.13	75.8
43210	16.07	14.41	14.12	22.09	36.2105
71522	12.37	8.44	13.82	14.68	15.9143
75721	25.21	30.02	30.61	41.07	78.2333
78331	15.01	12.03	11.97	17.13	78.2
78348	24.67	20.76	21.14	26.93	25.4667
78351	11.71	12.94	13.3	17.87	34.2667
Mean	18.43	18.38	18.00	24.47	48.611
Shaded cells	0	0	0	1	9

**Table 12:** Top 1% retrieval results for MUV dataset for descriptor (ECFC 4).

MUV Retrieval Result 1%	Previous Studies			Proposed Method
Activity Index	TAN	BIN	SQB	MLP
466	3.1	6.33	1.38	6.66667
548	8.62	14.89	11.38	28.6667
600	3.79	6.33	5.52	14.6667
644	7.59	11	8.97	14.6667
652	2.76	7	3.79	12
689	3.79	7.33	4.48	8
692	0.69	5.33	1.38	6.66667
712	4.14	8.22	5.17	8.66667
713	3.1	5.89	2.76	6
733	3.45	6.67	4.14	6
737	2.41	5.11	1.72	7.33333
810	2.07	6.78	1.72	6.66667
832	6.55	12.55	8.28	16.6667
846	9.66	13.11	12.41	16
852	12.41	13.78	9.66	18
858	1.72	5.11	1.38	7.33333
859	1.38	4.89	2.41	6.66667
Mean	4.542941	8.254118	5.091176	11.21569471
Shaded cells	0	2	0	12

**Table 13:** Top 5% retrieval results for MUV dataset for descriptor (ECFC 4).

MUV Retrieval Result 5%	Previous Studies			Proposed Method
Activity Index	TAN	BIN	SQB	MLP
466	5.86	10.44	8.62	12
548	22.76	27.22	24.14	46.6667
600	11.38	12.89	16.21	20.6667
644	17.59	19.67	17.93	25.3333
652	7.93	11.67	9.66	17.3333
689	9.66	13.22	11.72	15.3333
692	4.83	9.22	4.83	14.6667
712	10.34	16.45	11.03	14
713	7.24	9	5.86	12
733	8.97	10.11	8.62	9.33333
737	8.28	12	8.28	12
810	6.9	13.33	11.03	10
832	13.1	20.44	14.83	24.6667
846	28.62	26.11	26.9	36.6667
852	21.38	23.11	20	34.6667
858	5.86	9.11	6.21	14
859	8.97	9.44	8.62	11.3333
Mean	11.74529412	14.90765	12.61706	19.45098412
Shaded cells	0	4	0	13

**Table 14:** Top 1% retrieval results for DUD dataset.

DUD Retrieval Result 1%	Previous Studies		Proposed Method
Activity Index	TAN	SQB3	MLP
FGFR1T	2.5	2.92	3.17
FXA	1.92	3.36	1.64
GART	7.75	5.75	8.00
GBP	13.27	15.96	3.46
GR	2.31	3.21	3.08
HIVPR	3.55	3.55	5.16
HIVRT	1.63	1.86	5.00
HMGA	6.29	5.43	11.67
HSP90	1.62	4.05	4.21
MR	5.33	5.33	10.00
NA	2.24	5.31	5.20
PR	1.85	2.22	4.29
Mean	4.19	4.91	5.41
Shaded cells	0	4	8

**Table 15:** Top 5% retrieval results for DUD dataset.

DUD Retrieval Result 5%	Previous Studies		Proposed Method
Activity Index	TAN	SQB3	MLP
FGFR1T	6.67	7	8.17
FXA	7.88	8.29	7.95
GART	22.25	23.25	25.00
GBP	20.96	30.96	10.00
GR	6.41	8.46	7.69
HIVPR	11.77	11.29	13.87
HIVRT	4.88	6.98	9.09
HMGA	10.29	13.14	21.11
HSP90	8.11	8.38	13.68
MR	9.33	10	16.25
NA	5.1	9.8	10.00
PR	4.81	5.19	7.14
Mean	9.87	11.90	12.50
Shaded cells	0	3	9

**Table 16:** Ranking of enhanced Siamese multilayer perceptron method based on previous studies TAN, BIN, SQB, and SDBN using Kendall W test results.

DataSet	Retrieval Percentage	W	P	Rank Methods	
DS1	1%	0.593	0.00003	MLP	4.27
				SDBN	4.00
				BIN	3.27
				SQB	1.73
				TAN	1.73
	5%	0.588	0.000033	MLP	4.64
				SDBN	3.73
				BIN	2.82
				TAN	2.27
				SQB	1.55
DS2	1%	0.3673	0.0053	MLP	3.70
				BIN	3.65
				SDBN	3.40
				SQB	2.85
				TAN	1.40
	5%	0.34321	0.00821	BIN	4.15
				SQB	3.55
				SDBN	2.90
				MLP	2.70
				TAN	1.70
DS3	1%	0.698	0.0000129	MLP	4.60
				SDBN	4.10
				BIN	2.80
				SQB	1.90
				TAN	1.60
	5%	0.784	0.000002584	MLP	4.90
				SDBN	4.10
				SQB	2.10
				TAN	2.00
				BIN	1.90
MUV	1%	0.867	1.35 × 10^−9^	MLP	3.88
				BIN	3.12
				SQB	1.65
				TAN	1.35
	5%	0.702	8.24 × 10^−8^	MLP	3.74
				BIN	3.03
				SQB	1.82
				TAN	1.41
DUD	1%	0.3115	2.38 × 10^−2^	MLP	2.50
				SQB	2.08
				TAN	1.42
	5%	0.58333	0.0009118	MLP	2.67
				SQB	2.17
				TAN	1.17

## Data Availability

The MDL Drug Data Report (MDDR) dataset is owned by www.accelrys.com (accessed on 31 October 2021). A license is required to access the data. Maximum unbiased validation (MUV) datasets are freely available at http://www.pharmchem.tu-bs.de/lehre/baumann/MUV.html (accessed on 31 October 2021). The DUD dataset is freely accessible online as a benchmarking set at http://blaster.docking.org/dud/ (accessed on 31 October 2021). Software License: Python 3.7 in environment anaconda/Spyder was used with the following libraries: TensorFlow, Theano, Keras, Numpy, Pandas, and math. The license of statistics application (IBM spss) is licenseapp.utm.my.

## Supplementary Materials

The following are available online, Support Information which contains Table S1: The structure-activity classes of the MUV dataset, Table S2: The MDDR-DS1 structure activity classes, Table S3: The MDDR-DS2 structure activity classes, Table S4: The MDDR-DS3 structure activity classes, Table S5: DUD structure activity classes. Also Experiment Results of proposed method in each query with pruning in excel file.

## Sample Availability

Not available.
