# Supplementary material for: Similarity-Based Virtual Screen Using Enhanced Siamese Multi-Layer Perceptron
Source: Molecules. 2021 Nov 3;26(21):6669. doi: 10.3390/molecules26216669 (PMC8588560; doi:10.3390/molecules26216669)
Supplement: Supplementary file 1 [file molecules-26-06669-s001.zip › molecules-1405608-supplementary.pdf]

Supporting Information

Similarity Based Virtual Screen Using Enhanced Siamese Multi-Layer Perceptron

Mohammed Khaldoon Altalib[1], and Naomie Salim[1]

1- School of Computing, Universiti Teknologi Malaysia, Johor Bahru 81310, Malaysia.

Table Of Contents

Title	Page No.
**Table S1. The structure-activity classes of the MUV dataset**	3
**Table S2. The MDDR-DS1 structure activity classes**	4
**Table S3. The MDDR-DS2 structure activity classes**	5
**Table S4. The MDDR-DS3 structure activity classes**	6
**Table S5. DUD structure activity classes**	7

Supporting Information

Dataset's description

Two datasets have been used in this study:

The first dataset is Maximum Unbiased Validation (MUV) (freely available in https://pubmed.ncbi.nlm.nih.gov/19434821/) and the second dataset is the MDL Drug Data Report – MDDR (dataset is owned by www.accelrys.com. A license is required to access the data), and last dataset is Directory of Useful Decoys (DUD)

In the MUV dataset, there are 17 interaction groups in this dataset, with each class containing up to 30 active and 15,000 inactive molecules. The class composition for this dataset indicates that it involves classes with high diversity or more heterogeneous operations. Our research group in the previous articles has used this dataset. Table S1. explains MUV structure-activity classes

Table S1. The structure-activity classes of the MUV dataset

Activity Index	Activity Class	Pairwise Similarity	Sequence of activity molecules in file from - to	Sequence of inactivity molecules in file from - to	Number of active molecules
466	S1P1 rec. (agonists)	0.117	0-29	30-15030	30
644	Rho-Kinase2 (inhibitors)	0.122	0-29	30-15030	30
600	SF1 (inhibitors)	0.123	0-29	30-15030	30
689	Eph rec. A4 (inhibitors)	0.113	0-29	30-15030	30
652	HIV RT-RNase (inhibitors)	0.099	0-29	30-15030	30
712	HSP 90 (inhibitors) 30	0.106	0-29	30-15030	30
692	SF1 (agonists)	0.114	0-29	30-15030	30
733	ER-b-Coact. Bind. (inhibitors)	0.114	0-29	30-15030	30
713	ER-a-Coact. Bind. (inhibitors)	0.113	0-29	30-15030	30
810	FAK (inhibitors)	0.107	0-29	30-15030	30

737	ER-a-Coact. Bind. (potentiators)	0.129	0-29	30-15030	30
846	FXIa (inhibitors)	0.161	0-29	30-15030	30
832	Cathepsin G (inhibitors)	0.151	0-29	30-15030	30
858	D1 rec. (allosteric modulators)	0.111	0-29	30-15030	30
852	FXIIa (inhibitors)	0.150	0-29	30-15030	30
548	PKA (inhibitors)	0.128	0-29	30-15030	30
859	M1 rec. (allosteric inhibitors)	0.126	0-29	30-15030	30

The second dataset is the MDL Drug Data Report (MDDR). In this database, all molecules have been translated to the Pipeline Pilot(ECFC4 descriptor) and this database has been used by our research group. The MDDR contains three datasets. Table S2, Table S3, and Table S4. provide descriptions of all three datasets respectively.

1. MDDR-DS1: it contains 102516 molecules,11 activity groups, some of them with structurally homogenous active elements and others with structurally heterogeneous.
2. MDDR-DS2: it contains 102516 molecules,10 activity groups, The data collection consists of 10 homogeneous activity classes.
3. MDDR-DS3: it contains 102516 molecules,10 activity groups, The data collection consists of 10 heterogeneous activity classes.

Table S2. The MDDR-DS1 structure activity classes

Activity Index	Activity Class	Active molecules	sequence of activity molecules in file from - to	Pairwise similarity
**31420**	Renin inhibitors	1130	1 -1130	0.290
**71523**	HIV protease inhibitors	750	1131-1880	0.198
**37110**	Thrombin inhibitors	803	1881- 2683	0.180
**31432**	Angiotensin II AT1 antagonists	943	2684 - 3626	0.229

**42731**	Substance P antagonists	1246	3627 - 4872	0.149
**06233**	5HT3 antagonist	752	4873- 5624	0.140
**06245**	5HT reuptake inhibitors	359	5625 - 5983	0.122
**07701**	D2 antagonists	395	5984 - 6378	0.138
**06235**	5HT1A agonists	827	6379 - 7205	0.133
**78374**	Protein kinase C inhibitors	453	7206 - 7658	0.120
**78331**	Cyclooxygenase inhibitors	636	7659 - 8294	0.108

Table S3. The MDDR-DS2 structure activity classes

Activity Index	Activity Class	Active molecules	sequence of activity molecules in file from - to	Pairwise similarity
**07707**	Adenosine (A1) agonists	207	1 - 207	0.229
**07708**	Adenosine (A2) agonists	156	208 - 363	0.305
**31420**	Renin inhibitors	1130	364 -1493	0.290
**42710**	CCK agonists	111	1494 -1604	0.361
**64100**	Monocyclic - lactams □	1346	1605- 2905	0.336
**64200**	Cephalosporins	113	2906 - 3063	0.322
**64220**	Carbacephems	1051	3064 - 4114	0.269
**64500**	Carbapenems	126	4115- 4240	0.260
**64350**	Tribactams	388	4241 - 4628	0.305
**75755**	Vitamin D analogous	455	4629 - 5083	0.386

Table S4. The MDDR-DS3 structure activity classes

Activity Index	Activity Class	Active molecules	sequence of activity molecules in file from - to	Pairwise similarity
**09249**	Muscarinic (M1) agonists	900	1 - 900	0.111
**12455**	NMDA receptor antagonists	1400	901 - 2300	0.098
**12464**	Nitric oxide synthase inhibitors	505	2301 - 2805	0.102
**31281**	Dopamine -hydroxylase inhibitors	106	2806 - 2911	0.125
**43210**	Aldose reductase inhibitors	957	2912- 3868	0.119
**71522**	Reverse transcriptase inhibitors	700	3869- 4568	0.103
**75721**	Aromatase inhibitors	636	4569 - 5204	0.110
**78331**	Cyclooxygenase inhibitors	636	5205 - 5840	0.108
**78348**	Phospholipase A2 inhibitors	617	5841- 6457	0.123
**78351**	Lipoxygenase inhibitors	2111	6458 -8568	0.113

The last dataset used in this study is the Useful Decosys Directory (DUD), which was recently compiled for docking methods as a benchmark data set. It was introduced by Huang et al.,(2006) and recently used in both molecular and molecular virtual screening. Twelve DUD subsets with 704 active compounds and 25,828 decoys were used in this study as shown in Table S5.

Table S5. DUD structure activity classes

No.	***Dataset***	***Active and Inactive***	
		N_{dec}	N_a
1	*FGFR1T*	*4550*	*120*
2	*FXA*	*5745*	*146*
3	*GART*	*879*	*40*
4	*GBP*	*2140*	*52*

5	*GR*	*2947*	*78*
6	*HIVPR*	*2038*	*62*
7	*HIVRT*	*1519*	*43*
8	*HMGA*	*1480*	*35*
9	*HSP90*	*979*	*37*
10	*MR*	*636*	*15*
11	*NA*	*1874*	*49*
12	*PR*	*1041*	*27*
total		*25,828*	*704*

For training and testing the data, 50% of data used for training and 50% used for testing (because some classes have little molecules in the testing phase if used 20% or 30% of data; therefore, I used 50% of data for training and 50% for testing). The following table explains uploading files of datasets (DS1,DS2, and DS3)used for training and testing data. For the training phase, there are two input files and one output file. For the testing phase, there is one file as a testing dataset. The link for input and output files each dataset is here:

https://drive.google.com/drive/folders/1AVLI1SlL_stHC6E-FJZ0Kw_6H8AMtA_D?usp=sharing

which include:

- Excel file which contains the results of the experiment of MLP method in each dataset, and the results of the experiment of pruning.
